# Accelerated heparin-induced thrombocytopenia in a COVID-19 patient; a case report with literature review

**DOI:** 10.1016/j.amsu.2022.103749

**Published:** 2022-05-11

**Authors:** Hemin S. Mohammed, Fattah H. Fattah, Hawbash M. Rahim, Fahmi H. Kakamad, Shvan H. Mohammed, Rawezh Q. Salih, Abdulwahid M. Salih, Sharo Naqar

**Affiliations:** a Smart Health Tower, Madam Mitterrand Street, Sulaimani, Kurdistan, Iraq; b College of Medicine, University of Sulaimani, Madam Mitterrand Street, Sulaimani, Kurdistan, Iraq; c Kscien Organization, Hamdi Str, Azadi Mall, Sulaimani, Kurdistan, Iraq; d Gaziosmanpasa University, Faculty of Medicine, Tokat, Turkey

**Keywords:** Heparin-induced thrombocytopenia, COVID-19, Unfractionated heparin, Platelet

## Abstract

**Introduction:**

Heparin-induced thrombocytopenia (HIT) is a rare and serious immune-mediated complication of heparin therapy which is seldom reported in association with COVID-19. This report aims to present a case of accelerated HIT in a severe COVID-19 patient.

**Case report:**

A 63-year-old man presents with symptoms of COVID-19 for one week. He was conscious, ordinated, feverish, and had diffused chest crackles. Initial laboratory tests revealed elevated C-reactive protein of 87.66 mg/dL, elevated D-dimmer of 1258.9 ng/ml, elevated ferritin of 1020 ng/ml, and his platelet count was within the normal range. Polymerase chain reaction (PCR) confirmed the diagnosis of COVID-19. On the 9th day of admission, he developed a progressive worsening of dyspnea. His D-dimmer level significantly increased to 7020 ng/ml, and his interleukin-6 was 27.3 pg/ml. Hence, we started him on unfractionated heparin (UFH) for thromboprophylaxis. On the 12th day of hospitalization, the platelet count dropped from 258000 to 111000 cells/μL. He had a high probability of HIT (4Ts score = 6). As a result, we discontinued UFH and switched him to apixaban. His platelet count normalized (174000 cells/μL) within two weeks of ceasing UHF.

**Discussion:**

HIT results from the production of antibodies against platelet factor 4/heparin complexes. It is associated with a diminished platelet count within 5–10 days post heparin initiation. Because thrombocytopenia can occur in COVID-19 patients, HIT is seldom suspected.

**Conclusion:**

HIT should be considered a differential diagnosis in COVID-19 patients with thrombocytopenia.

## Highlights

• Heparin-induced thrombocytopenia is a dangerous immune-mediated complication of heparin therapy.
• It has been rarely reported in association with COVID-19.
• This report presents a case of accelerated heparin-induced thrombocytopenia in a severe COVID-19 patient.

## 1 Introduction

The etiologic agent for COVID-19 infection is the severe acute respiratory syndrome coronavirus 2 (SARS-CoV-2) [1]. The virus was initially discovered in China in late 2019, and it has since aggressively spread around the globe, impacting every country [2]. In March 2020, the World Health Organization (WHO) officially declared COVID-19 a pandemic [3]. This pathogenic condition is highly infectious and circulates primarily through coughing, talking within close proximity, or sneezing [4]. Many infected COVID-19 patients have mild symptoms (such as loss of taste or smell, fever, fatigue, and dry cough) or none at all. However, acute respiratory distress syndrome (ARDS), a potentially deadly ailment, can occur in roughly 14% of patients [5]. ARDS is more likely to occur in people predisposed to specific risk factors, such as diabetes, old age, and hypertension [6]. Although it is generally considered a respiratory syndrome, COVID-19 is recognized as a system disorder since it may involve multiple organs of the body, and this might be due to the high concentration of angiotensin-converting enzyme 2 in many organs [7]. COVID-19 is commonly associated with hyperco agulability and thromboembolic complications, particularly in severe cases. These patients frequently require anticoagulation medication, such as heparin, for thromboprophylaxis [8,9]. Heparin-induced thrombocytopenia (HIT) is a rare and dangerous immune-mediated complication of heparin treatment that results from the production of antibodies against platelet factor 4 (PF4)/heparin complexes [10]. It is associated with an increased risk of thrombosis and a decreased platelet count. Even though rare, HIT has been reported in COVID-19 patients with an unknown true prevalence [11].

The current report aims to present a case of accelerated HIT in a severe COVID-19 patient while taking the SCARE 2020 guidelines into consideration in the preparation of this paper [12].

## 2 Case presentation

**Patient information**: A 63-year-old man presents with fever, shortness of breath, cough, myalgia, and generalized body ache for one week and gets admitted to our hospital. The patient's oxygen saturation was 87% at room temperature. He had no past medical or surgical history.

**Clinical findings**: The patient was conscious, ordinated, febrile, and had diffused chest crackles.

**Diagnostic approach**: Initial laboratory tests revealed elevated C-reactive protein levels of 87.66 mg/dL (normal range 5 mg/dL), elevated D-dimmer levels of 1258.9 ng/ml (normal range 500 ng/ml), and elevated ferritin levels of 1020 ng/ml (normal range 30–400 mg/dL). Platelet count and renal and liver function tests were relatively within normal limits. High-resolution computed tomography was performed and showed bilateral interstitial opacities, which indicated moderate to severe COVID-19. A reverse transcriptase-polymerase chain reaction was performed and confirmed the diagnosis of COVID-19.

**Therapeutic intervention**: On the 9th day of admission, the patient developed a progressive worsening of dyspnea. His D-dimmer level significantly increased to 7020 ng/ml, and his interleukin-6 was 27.3 pg/ml. Hence, we started unfractionated heparin (UFH) for thromboprophylaxis. On the 12th day of hospitalization, the platelet count dropped from 258000 to 111000 cells/μL. He had a high probability of HIT (4Ts score = 6). As a result, UFH was discontinued and switched to a direct thrombin inhibitor (apixaban 5 mg), which was given twice a day for one week, and then 5 mg was continued for two months.

**Follow-up and outcome**: The patient's total platelet count normalized (174000 cells/μL) within two weeks of discontinuing UHF.

## 3 Discussion

Severe cases of COVID-19 are associated with the disturbance of the immune system and thromboembolic complications due to endothelial damage and coagulation system activation [9]. The incidence of thrombosis is high in COVID-19 patients, with 30% of patients having pulmonary and deep vein thrombosis [13]. Heparin is widely used as prophylaxis against thromboembolic events in COVID-19 patients as it confers survival benefits. HIT is a rare complication of heparin treatment that develops due to the production of autoantibodies (immunoglobulin G) against heparin exposure, more specifically, against PF4/heparin complexes. This leads to platelet activation and initiates the coagulation cascade that results in systemic clot formation and platelet depletion, which can further complicate the COVID-19 condition [10]. The factors associated with an increased risk are extracorporeal membrane oxygenation, surgery, hemodialysis, UFH, or low molecular-weight heparin (LMWH) [14]. UFH has ten times the tendency to cause HIT relative to LMWH [15]. There are currently a few reports in the literature describing the occurrence of HIT in COVID-19 patients [11].

HIT incidence is 0.2–0.45% in the general population, which increases to 2.7% in critically ill patients [10,16]. Meanwhile, the exact prevalence of HIT in COVID-19 is currently unknown, and the literature is conflicting regarding this aspect [10]. Some studies have reported a low prevalence, while others have reported a high prevalence [13]. Patell and associates detected HIT antibodies at 25 days in 12% of COVID-19 patients given UFH; however, their results were not confirmed by the serotonin release assay (SRA) [17]. Daviet et al. reported an incidence of 8% [11]. Meanwhile, Bidar et al. reported an incidence of only 4.3% [18].

According to the available studies in the literature, most occurrences of HIT in COVID-19 are in patients older than 50 years, with a much higher male predominance [11,13], as was the current case. Patients with HIT can be asymptomatic or associated with life-threatening thrombosis. Bidar and colleagues reported two incidences of HIT in COVID-19, with one of them lacking symptoms and the other associated with thrombosis [18].

HIT is associated with a decreased platelet count by nearly 40–50%, occurring within 5–10 days post heparin initiation. Thrombocytopenia has also been reported in up to 55% of COVID-19 patients, with a lower platelet count in more severe cases [19]. Hence, HIT is rarely suspected as a differential diagnosis in COVID-19 patients [20].

The initial diagnosis of HIT is based on clinical suspicion. According to the American Society of Hematology guidelines, the 4Ts score can be used to estimate the probability of HIT [19]. Although the presence of PF4/heparin antibodies is a hallmark of HIT, these antibodies have also been observed in COVID-19 patients [13]. Additionally, viral and bacterial infections and autoimmune responses can also result in the production of anti-PF4/heparin antibodies [15]. HIT can be diagnosed via immunoassays to detect anti-PF4/heparin antibodies in cases with a moderate to high 4Ts score. Other functional assays that can be used to confirm the diagnosis include the heparin-induced platelet aggregation assay and SRA. However, in cases with high 4Ts scores, confirmatory tests are not required [10].

The management of thrombotic complications due to COVID-19 and HIT is quite different. In COVID-19, heparin is used as the primary antithrombotic agent. Meanwhile, the immediate cessation of heparin and switching to an alternative agent is the mainstay of management in HIT. In most patients, this results in platelet count normalization within seven days [10]. However, switching to other anticoagulants is difficult as they can increase the risk of bleeding, are more expensive, and are hard to monitor [21]. If HIT is left untreated, it can lead to the development of other thrombotic complications in 30–75% of patients, and even death in 10% [20]. Patell and associates reported that three out of five HIT patients with COVID-19 developed hemorrhage after the move from heparin to direct thrombin inhibitors [17]. In another study, Riker et al. treated three patients successfully by switching to bivalirudin [16]. Lingamaneni et al. switched to argatroban, but unfortunately, their case died a day later [19]. Madala et al. switched to apixaban and successfully treated their patient [22]. In this study, apixaban was used as an alternative to heparin, which was associated with a good outcome.

Our study had its limitations as it lacked definitive diagnosis using functional and immunological assays due to limited resources at our hospital. However, confirmatory tests are not mandatory in patients with a high-probability 4Ts score of 6–8 [15]. Despite the limitations, our study indicated the occurrence of HIT in a severe COVID-19 patient based on clinical suspicion and rapid elevation of platelet count after heparin cessation.

In conclusion, HIT is a rare thrombotic complication of heparin treatment. COVID-19 can be an independent risk factor for HIT development. Hence, HIT should be a differential diagnosis in COVID-19 patients with thrombocytopenia. Switching from heparin to direct thrombin inhibitors should be done under close monitoring. More extensive research is needed to determine the true prevalence of HIT in COVID-19.

## Consent

Written informed consent was obtained from the patient and the patient's family for publication of this case report and accompanying images. A copy of the written consent is available for review by the Editor-in-Chief of this journal on request.

## Provenance and peer review

Not commissioned, externally peer-reviewed.

## Ethical approval

Approval is not necessary for case report (till 3 cases in single report) in our locality.

The family gave consent for the publication of the report.

## Sources of funding

None is found.

## Author contribution

Abdulwahid M. Salh: major contribution of the idea, final approval of the manuscript. Hemin S. Mohammed: physician the managing case, final approval of the manuscript. Fahmi H. Kakamad, Hawbash M. Rahim: Writing the manuscript, literature review, final approval of the manuscript. Shvan H. Mohammed, Rawezh Q. Salih, Sharo Naqar, Fattah H. Fattah: literature review, final approval of the manuscript.

## Trial registry number

Not applicable.

## Guarantor

Fahmi Hussein Kakamad.

Fahmi.hussein@univsul.edu.iq.

## Declaration of competing interest

None to be declared.
